# Patient-Related Characteristics Associated with Treatment Modifications and Suboptimal Relative Dose Intensity of Neoadjuvant Chemotherapy in Patients with Breast Cancer—A Retrospective Study

**DOI:** 10.3390/cancers15092483

**Published:** 2023-04-26

**Authors:** Eva Kjeldsted, Julie Gehl, Dina Melanie Sørensen, Alexey Lodin, Silvia Gonzalez Ceballos, Susanne Oksbjerg Dalton

**Affiliations:** 1 Danish Research Center for Equality in Cancer (COMPAS), 4700 Næstved, Denmark; 2 Department of Clinical Oncology and Palliative Care, Zealand University Hospital, 4700 Næstved, Denmark; 3 Survivorship and Inequality in Cancer, Danish Cancer Society Research Center, 2100 Copenhagen, Denmark; 4 Department of Clinical Medicine, Faculty of Health and Medical Sciences, University of Copenhagen, 2200 Copenhagen, Denmark; 5 Aleris Hospital Ringsted, 4100 Ringsted, Denmark

**Keywords:** breast neoplasms, neoadjuvant therapy, guideline adherence

## Abstract

**Simple Summary:**

This study aimed to examine clinical practice patterns of adherence to the planned neoadjuvant chemotherapy doses, patient-related characteristics associated with treatment modifications and tumour response in patients with breast cancer. Most patients received their chemotherapy without dose reductions or delay, but still one out of four patients received a suboptimal chemotherapy dose intensity. Age ≥ 65 years, comorbidity, taking long-term medications and being overweight were significantly associated with dose reductions, dose delays, discontinuation or suboptimal chemotherapy dose intensity. Increased focus on developing and testing supportive care initiatives to encompass patients of older age or with chronic diseases may contribute to reduce side effects and improve chemotherapy adherence.

**Abstract:**

Background: Reduced relative dose intensity (RDI) of neoadjuvant chemotherapy (NACT) in patients with breast cancer may compromise treatment outcome and survival. We examined patient-related characteristics associated with treatment modifications and suboptimal RDI and tumour response in patients with breast cancer. Methods: In this observational study, electronic medical records were reviewed retrospectively for female patients with breast cancer scheduled for NACT at a university hospital in Denmark between 2017 and 2019. The RDI (ratio of delivered dose intensity in relation to standard dose intensity) was calculated. Multivariate logistic regression analyses examined associations of sociodemographics, general health and clinical cancer characteristics with dose reductions, dose delays, discontinuation of NACT and suboptimal RDI < 85%. Results: Among 122 included patients, 43%, 42% and 28% experienced dose reductions, dose delays ≥3 days and discontinuation, respectively. A total of 25% received an RDI < 85%. Comorbidity, taking long-term medications and being overweight were statistically significantly associated with treatment modifications, while age ≥ 65 years and comorbidity were associated with RDI < 85%. Around one third of all patients had radiologic (36%) or pathologic (35%) complete tumour response, with no statistically significant differences by RDI < or ≥85% irrespective of breast cancer subtype. Conclusions: While most patients had RDI ≥85%, still one out of four patients received an RDI < 85%. Further investigations of possible supportive care initiatives to improve patients’ treatment tolerability are needed, particularly among subgroups of older age or with comorbidity.

## 1. Introduction

Breast cancer is the most common cancer among women worldwide with an estimated 2.26 million incident breast cancer cases in 2020 [1]. Most patients with breast cancer receive curatively intended treatment consisting of surgery and adjuvant chemotherapy (ACT), endocrine therapy, human epidermal growth factor 2 (HER2)-targeted therapy, bisphosphonate or radiotherapy depending on the breast cancer subtype and surgery result [2]. Patients with breast cancer presenting with large primary tumours and/or axillary lymph-node metastases are treated with neoadjuvant chemotherapy (NACT) before surgery, which has shown to be effective in downsizing/downstaging tumours and thereby increasing the possibility for breast-conserving and axillary-sparing procedures [3].

Maintaining a high dose intensity of chemotherapy is important to achieve the full benefit of the treatment [4]. Nevertheless, treatment modifications may be necessary to manage chemotherapy-related toxicities [5,6]. The relative dose intensity (RDI), defined as the ratio of delivered dose intensity in relation to the standard dose intensity (range 0–100%), is a well-established measure of patient tolerability and adherence to chemotherapy taking into account the extent of dose reductions and dose delays [6]. In general, an RDI ≥ 85% for chemotherapy has been suggested as a meaningful cut-off for sufficient chemotherapy dosing among patients with breast cancer as chemotherapy response below this threshold may be suboptimal in regard to disease-free and overall survival [7,8,9]. 

Previous research on treatment modifications of ACT among patients with breast cancer found that factors such as older age, body surface area (BSA) > 2.0 m^2^, comorbidities, lower functional status, anthracycline-based regimens and febrile neutropenia were statistically significantly associated with dose reductions and/or dose delays [10,11,12,13]. Furthermore, older age, febrile neutropenia and severe hypersensitivity reactions to taxanes [10,14] and larger visceral or intramuscular adiposity [15] were predictors of an RDI < 85% of ACT. Less is known about the extent of and reasons for treatment modifications of NACT in patients with breast cancer despite the fact that patients eligible for NACT have a locally advanced disease stressing the need for maintaining dose intensity. Among a cohort of 237 patients with breast cancer from the United States, nearly a quarter (24%) received an RDI of NACT < 85%. The most common toxicities requiring dose delays and reductions were myelosuppression, infection and neuropathy [16].

It is of high clinical relevance to develop strategies of early identification of the vulnerable patients with breast cancer at risk of unplanned reductions in dose intensity of NACT. An important first step is to understand the clinical practice patterns of treatment modifications of NACT including the characteristics of the patients who may be less likely to maintain a high dose intensity and, thus less likely to have a positive treatment response. The aim of this study was first to examine the incidence of and reported reasons for dose reductions, dose delays and discontinuation of NACT among patients with breast cancer in a Danish regional setting. Secondly, to investigate the associations between patient-related characteristics and treatment modifications as well as suboptimal RDI < 85%. Lastly, we described the patients’ tumour response to NACT.

## 2. Materials and Methods

### 2.1. Study Design and Setting

Before study initiation, we obtained permission from the Danish Patient Safety Authority (number 3-3013-3055/1) to access the medical records, and the study was listed on the register for the processing of personal data in research in Region Zealand (number REG-057-2019). To identify patients for this retrospective observational study, we reviewed the electronic medical records of all patients with breast cancer (n = 669) who were referred to the outpatient clinic at the Department of Clinical Oncology and Palliative Care, Zealand University Hospital, Næstved, Denmark between 25 November 2017 and 31 May 2019. The department provides oncological care to patients with breast cancer living in Region Zealand (around 840,000 inhabitants). Patients met the inclusion criteria for this study if having newly diagnosed histologically verified invasive breast cancer and planned to start NACT based on the Danish Breast Cancer Cooperative Group guidelines [17,18].

### 2.2. Neoadjuvant Chemotherapy

According to the Danish Breast Cancer Cooperative Group guidelines introduced in 2016 and applicable to patients in this study [17], the standard NACT consisted of anthracycline and taxane-based regimens. The treatment was a combination of three to four cycles of epirubicin (90 mg/m^2^) and cyclophosphamide (600 mg/m^2^) administered at a three-week interval followed by three to four cycles of paclitaxel (80 mg/m^2^) with nine to twelve weekly doses administered on day 1, 8 and 15. Furthermore, HER2 positive patients were offered treatment with trastuzumab and pertuzumab. During NACT, patients were evaluated by clinical breast palpation and magnetic resonance imaging (MRI) at regular intervals typically before NACT (week 0), after two chemotherapy cycles (week 6) and before surgery (week 21/24). Additional scans in between were added based on the individual treatment response. In case of no response after two chemotherapy cycles, patients were typically converted to paclitaxel according to guidelines. Additional treatment modifications could be decided based on toxicity or other critical reasons.

### 2.3. Data Extraction

Medical records data were extracted retrospectively based on a data extraction form with pre-defined variables to ensure consistency. Data included demographic and social characteristics (age, cohabitation status, work market affiliation), lifestyle (smoking, alcohol consumption), general health (kidney function, comorbidity and prescription medication), Body Mass Index (BMI) and WHO performance status at time of breast cancer diagnosis as well as clinical information on tumour characteristics, planned and received NACT. All reasons for treatment modifications documented by the oncologists were extracted but only the main/most severe reason was counted. The reasons for treatment modifications were grouped into toxicity, comorbidity, patient request (non-medical reasons of convenience, e.g., holidays), administrative reasons (treatment plan errors), allergic reactions (infusion-related hypersensitivity reactions to chemotherapy) and unknown (not reported). Due to inconsistent reporting of the Common Terminology Criteria for Adverse Events (CTCAE) for toxicity symptoms, these were not extracted. After data extraction, interrater reliability was evaluated through the reassessment of a subsample (≈10%) [19].

### 2.4. Outcomes

We defined treatment modifications to NACT as follows: Dose reductions at cycle 1 were planned initially by the oncologist. Dose reductions after cycle 1 included unplanned reductions during NACT (could happen one or more times per patient). Dose delays were registered if doses were given ≥ 3 days later than the originally scheduled treatment date, and only if it resulted in the postponement of the next planned chemotherapy dose (could happen one or more times per patient). Discontinuation was registered if NACT was stopped earlier than planned and the patient was referred to surgery. 

The RDI in percent was calculated for each patient based on methods used by Weycker et al. [6]. RDI was defined as the amount of drug administered per unit of time (=delivered total dose in mg/m^2^ divided by actual time used to complete treatment in a number of weeks) as a fraction of the standard amount of drug per unit of time (=standard total dose in mg/m^2^ divided by standard time to complete treatment in a number of weeks based on the Danish Breast Cancer Cooperative Group guidelines [17]). The treatment weeks were counted as the number of weeks between the first dose of chemotherapy in the first cycle and the last dose of chemotherapy in the last cycle. The RDI for each chemotherapy drug was calculated separately and the total RDI was calculated by taking the average of all drugs [6]. An RDI < 85% was considered suboptimal [7,8,9].

Tumour size change was registered as the maximum diameter of the largest lesion in millimeter (mm) from the sequential MRIs. Based on the last performed MRI before surgery, radiologic complete response (rCR) was defined as tumour size = 0 mm/disappearance of all lesions, and radiologic non-complete response (non-rCR) as tumour size > 0 mm/residual tumour. 

Treatment response in surgically excised breast tissue was registered from the pathology report described according to the Danish Breast Cancer Cooperative Group guidelines and using a modified version of the Miller–Payne classification [17]: At pathologic response grade I/pathologic complete response (pCR), no remaining malignant cells could be identified from the removed tissue (ductal carcinoma in situ could be present). Grades II–IV (non-pCR) were divided based on the percentage of remaining malignant cells. Grade II: more than 90% reduction in tumour cells; Grade III: between 30 and 90% reduction in tumour cells; Grade IV: less than 30% reduction in tumour cells.

### 2.5. Statistical Analyses

Descriptive statistics were used to assess the distribution of patient characteristics, treatment modifications and treatment response. Median and range were used for RDI and tumour sizes as these were not normally distributed. Tumour response was stratified by breast cancer subtype (estrogen receptor [ER]+HER2 normal, ER+HER2+, ER-HER2 normal, ER-HER2+), and the Fisher’s exact test was used to compare radiologic or pathologic response by RDI< or ≥85% for each subtype. Logistic regression analyses examined the independent associations between categorical covariates (age, cohabitation status, work market affiliation, BMI, BSA, comorbidity, long-term medications, breast cancer subtype, tumour size, positive lymph nodes) and the four binary outcomes related to treatment modifications of NACT: (1) dose reductions (yes vs. no), (2) dose delays (yes vs. no), (3) discontinuation (yes vs. no), and (4) RDI (< vs. ≥85 %). Analyses were adjusted for age (continuous variable) and estimated Glomerular Filtration Rate (eGFR) (<90, ≥90 mL/min/1.73 m^2^) as confounders and use of granulocyte-colony stimulating factor (G-CSF) during NACT (yes, no) as a mediator. Associations were estimated as odds ratios (OR) with 95% confidence intervals (CI), and *p*-values less than 0.05 were considered statistically significant. Regression analyses showing associations between RDI and tumour response or pCR were not meaningful to conduct due to the small strata. The missing data corresponding to <5% (work market affiliation n = 3, tumour size change n = 4, pathological response grade = 2) were omitted from the analyses. All analyses were performed in RStudio Statistical Package version 1.4.110 for Windows 6 (RStudio, Boston, MA, USA).

## 3. Results

### 3.1. Descriptive Characteristics 

**Figure 1:**
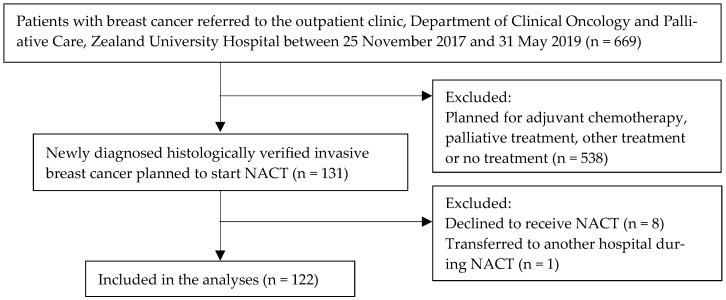
Flow chart of included patients.

Of the 669 patients with breast cancer who were referred to the oncological outpatient clinic, 538 patients were scheduled for ACT, palliative treatment, other treatment or no treatment. The remaining 131 patients met the inclusion criteria for this study and were planned to start NACT. After excluding patients who declined to receive NACT (n = 8) or who were transferred to another hospital during treatment (n = 1), data from 122 female patients were included in the analyses (Figure 1).

The median age at diagnosis was 50 years (range 25–78 years) and the majority were living with a partner (70%), working (58%), or on age retirement (24%). Most had performance status 0 (97%), were never or previous smokers (68%), had no comorbidities (66%) and received no or 1–4 different long-term medications (both 44%) (Table 1). Details of comorbidity and long term-medications at diagnosis are available in Supplementary Table S1. The median BMI was 26 kg/m^2^ (range 17–45 kg/m^2^). Most patients had breast cancer subtype ER+HER2- (48%) and positive lymph nodes at the time of diagnosis (56%). All patients received anthracycline and taxane-based chemotherapy regimens and were initially planned for six cycles corresponding to 18 weeks (48%) or eight cycles corresponding to 24 weeks (50%), respectively (Table 1).

### 3.2. Treatment Modifications and Relative Dose Intensity 

**Figure 2:**
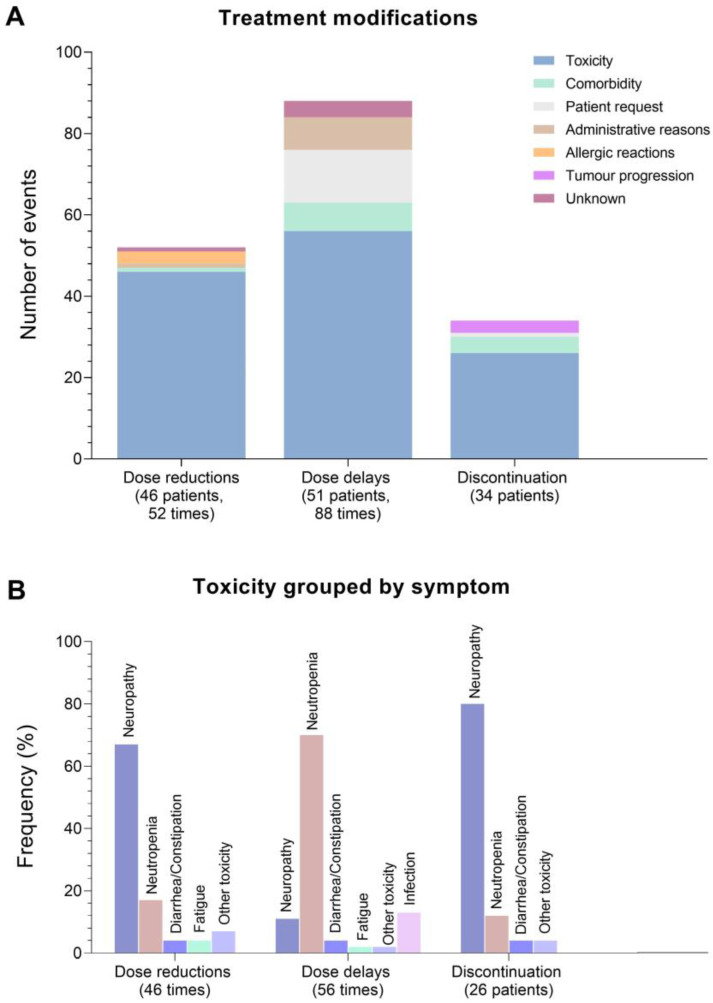
Incidence of and reasons for treatment modifications (unplanned dose reductions, dose delays and discontinuation) of neoadjuvant chemotherapy among 122 patients with breast cancer between 2017 and 2019. (**A**) Modifications and reasons illustrated as number of times since some patients experienced more than one dose reduction and dose delay for different reasons. (**B**) Toxicity symptoms for unplanned dose reductions, dose delays and discontinuation of neoadjuvant chemotherapy in percent.

In total 53 patients (43%) had dose reductions either planned at cycle 1 due to older age/comorbidity (n = 10), unplanned after cycle 1 (n = 46) or both (n = 3). Among the 46 patients with unplanned dose reductions, doses were reduced 52 times. A total of 51 patients (42%) had 88 dose delays of ≥3 days one or more times with a total median delay of 8 days (range 3 to 32 days). Furthermore, 34 patients (28%) had discontinuation of NACT. The large majority of reported reasons were toxicity, comorbidity and patient request (Figure 2A). More specifically, neutropenia and neuropathy were the most frequent toxicity symptoms, with neutropenia accounting for 70% of dose delays and neuropathy for 67% and 80% of dose reductions and discontinuation related to toxicity, respectively (Figure 2B). The median RDI was 95.23% (range 8.33% to 100%). In total 25% of patients (n = 30) received a suboptimal RDI < 85% (Table 2). Supplementary Figure S1 illustrates the planned and received treatment of the 30 patients with RDI < 85%. During NACT, 31% of patients (n = 38) received G-CSF corresponding to one (n = 20), two (n = 11) or three times (n = 7).

In the multi-variate logistic regressions of the associations between patient-related characteristics and treatment modifications, having one comorbidity (OR 3.4, 95% CI 1.3–8.9), taking 1–4 (OR 4.1, 95% CI 1.6–10.8) or ≥5 (OR 8.4, 95% CI 1.8–39.9) different long term medications were statistically significantly associated with increased odds of dose reduction. Furthermore, having positive lymph nodes at diagnosis was statistically significantly associated with dose delay (OR 2.3, 95% CI 1.0–5.6). With regards to the discontinuation of NACT, odds were statistically significantly increased with being overweight (OR 2.8, 95% CI 1.1–7.4), ≥2 comorbidities (OR 4.8, 95% CI 1.0–22.8) or ≥5 different long term medications (OR 8.2, 95% CI 1.9–34.3). Lastly, when investigating RDI, age ≥ 65 years (OR 3.0, 95% CI 1.1–8.7) and ≥2 comorbidities (OR 5.1, 95% CI 1.0–26.4) were statistically significantly associated with the increased odds of receiving an RDI < 85% (Table 3).

### 3.3. Tumour Response to Neoadjuvant Chemotherapy

At the last performed MRI before surgery, 36% of patients (n = 43) had rCR, and after surgery, 35% (n = 41) had pCR. Patients with subtype ER-HER2+ had rCR and pCR rates of 78% and 83%, respectively, and ER+HER2 normal had rCR and pCR rates of 17% and 12%, respectively. There were no statistically significant differences in rCR or pCR by RDI< or ≥85% irrespective of subtype (all *p* > 0.05) (Table 4).

**Figure 3:**
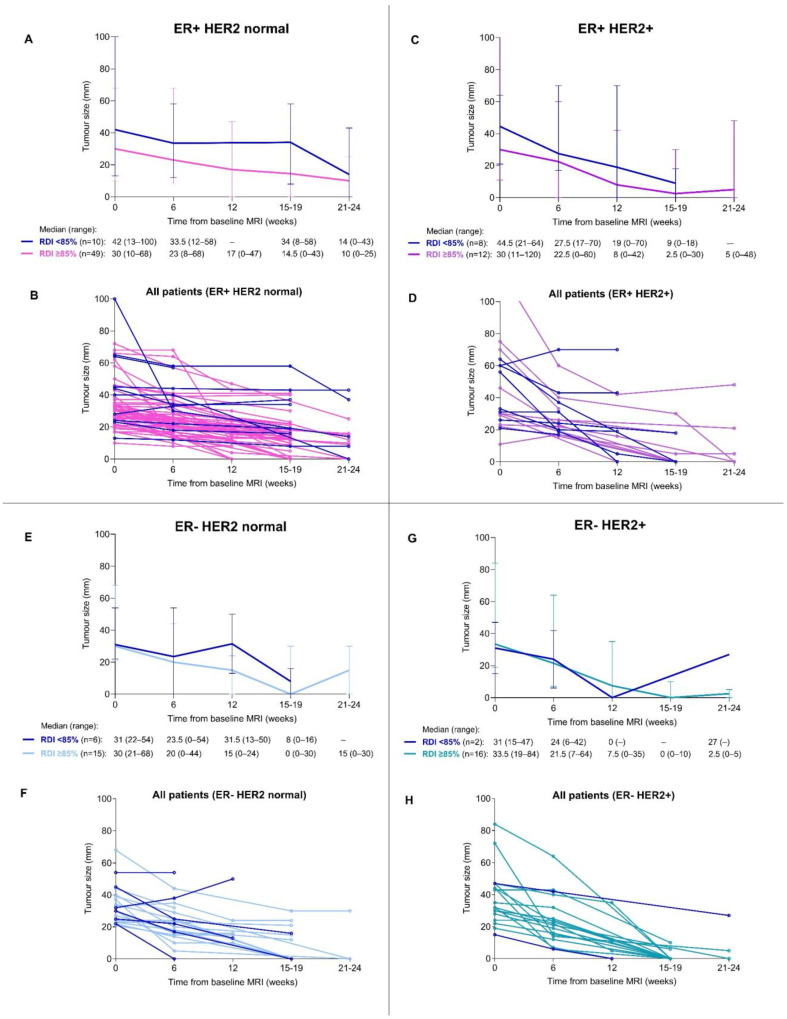
Tumour size change in millimeters measured by MRI in 118 patients with breast cancer receiving neoadjuvant chemotherapy between 2017 and 2019, by subtype ER+HER2 normal, ER+HER2+, ER-HER2 normal and ER-HER2+, respectively. (**A**,**C**,**E**,**G**) Median and range for tumour sizes by RDI (≥85% vs. <85%). (**B**,**D**,**F**,**H**) Tumour size changes for each patient by RDI (≥85% vs. <85%). Abbreviations: ER, estrogen receptor; HER2, human epidermal growth factor receptor 2; MRI, magnetic resonance imaging; RDI, relative dose intensity.

**Figure 4:**
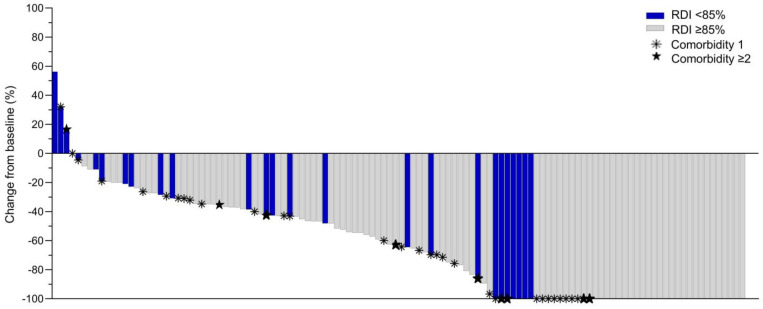
Tumour size change from baseline to time of surgery in percent measured by magnetic resonance imaging in 118 patients with breast cancer between 2017 and 2019, by RDI of neoadjuvant chemotherapy (<85% or ≥85%) and comorbidity (1 or ≥2 chronic diseases). Abbreviations: RDI, relative dose intensity.

Median tumour size changes from baseline to surgery grouped by breast cancer subtype and received RDI are illustrated in Figure 3A,C,E,G and are further stratified for each patient with the respective subtype and RDI in Figure 3B,D,F,H. Variations in response appeared according to subtype and RDI< or ≥85% but without examinations for statistically significant differences. Tumour response is further elaborated in Figure 4 showing tumour size change in percent for each patient with number of comorbidities and received RDI. Among the 43 patients with 100% tumour reduction (rCR), the majority seemingly had no comorbidity and received RDI ≥85% but without examinations for statistically significant differences.

## 4. Discussion

This study showed that dose delays, dose reductions and discontinuation of NACT were common for patients with breast cancer in a real-world setting. Although most patients received an RDI ≥85%, still one quarter received an RDI < 85%. Age ≥ 65 years, comorbidity, taking long-term medications and being overweight were patient-related characteristics statistically significantly associated with reduced chemotherapy delivery. These findings indicate that subgroups of older age or with concurrent chronic diseases requiring prescription medications may be particularly susceptible to receiving a suboptimal RDI. Around one third of all patients had rCR or pCR. There were no statistically significant differences in rCR or pCR by RDI regardless of breast cancer subtype. 

Dose reductions and dose delays occurred in around 40% and discontinuation in nearly 30% of the patients in our study. Recent findings of dose delay and reductions in patients with breast cancer were between 24% and 48% [5,6,10], but different definitions, disease stage and chemotherapy regimens challenge direct comparison. For instance, whereas others included dose delays of ≥7 days [5,6,10], we believed that a cut-off of ≥3 days was clinically meaningful since it would delay the next chemotherapy dose, and more short delays could result in a longer total delay potentially postponing surgery. We found that neutropenia was the most frequent reason for dose delay. This is not surprising as chemotherapy-induced neutropenia remains a major toxicity symptom to myelosuppressive chemotherapeutic agents [20]. Administration of G-CSF is used commonly as supportive care to prevent or treat chemotherapy-induced neutropenia [20,21]. G-CSF use has been associated with an increased likelihood of maintaining a high RDI [10,22]. In a retrospective study from Canada examining clinical practice patterns of G-CSF use between 2008 and 2019, 57% of 6662 patients receiving NACT or ACT for breast cancer were prescribed G-CSF at some point during treatment. Most patients, however, only received G-CSF once [23]. In our study, 31% of patients received G-CSF and, in most cases, only once. This seems low compared to the number of registered neutropenic events delaying chemotherapy doses. We did not make sub-analyses of G-CSF use, but others found that patients with breast cancer aged ≥ 65 years were less likely to receive this prophylactic supportive care compared to younger patients [10,23]. Neuropathy accounted for most of both dose reductions and discontinuation of NACT in our study, which corresponds to peripheral neuropathy being a common toxicity symptom to taxane-based chemotherapy [24]. This is similar to neuropathy being the most frequent reason (33%) for doses of NACT adjusted or missed among 243 patients with breast cancer from the United States [25]. Of note, patient request for non-medical reasons accounted for 15% of dose delays in both our study and the cohort of patients with breast cancer receiving NACT by Usiskin et al. [16]. This highlights the importance of communicating to patients and their relatives that unnecessary delays should be minimized to maintain dose intensity.

In our study, 25% of patients received an RDI < 85% corresponding to previous findings of 24% in patients receiving NACT for breast cancer [16]. Other recent studies on chemotherapy dose intensity and breast cancer combining NACT and ACT found similar rates of RDI < 85% of 21% [26], between 25–30% [6,10,22,27] and 39% [5]. An RDI ≥ 85% of anthracycline and taxane-based regimens has been considered the optimal cut-off for optimizing overall survival benefits [7,8,9]. However, Zhang et al. [22] demonstrated a possible variation of the optimal cut-off to improve cause-specific and overall survival by breast cancer subtype (RDI ≥ 85% for subtype ER+/progesterone+/HER2-, and RDI ≥ 75% for subtype ER-/progesterone-/HER2- [triple negative]) in a retrospective study with 674 patients from the United States receiving NACT or ACT. Likewise, Qi et al. [27] suggested a cut-off of RDI ≥ 85% or ≥80% to improve disease-free and overall survival for ER+ patients and ER- patients, respectively, based on a retrospective analysis of 293 patients with breast cancer from Western China. Both studies, however, also underlined a need for further investigations in more geographically diverse patient populations of details on separate effects of dose reductions or dose delays, optimal regimen-specific RDIs, optimal NACT vs. ACT RDIs and optimal RDI in HER2+ patients [22,27]. The goal of maintaining a high RDI of chemotherapy and the fact that a higher RDI also increases the probability of toxicity remains a challenging dilemma to clinical practice. In patients experiencing severe toxicity symptoms negatively affecting quality of life, oncologists must compromise dose intensity and introduce treatment modifications to minimize harm. Furthermore, challenges associated with adequate chemotherapy dosing of the heterogeneous breast cancer population continue. On the one hand, initially planned dose reductions based on, i.e., older age or comorbidity, or empirically capped BSA at 2.0 m^2^ for chemotherapy dosing in patients with severe obesity, are individual patient evaluations introduced in attempt to limit excess toxicity or overdosing and improve treatment tolerability. On the other hand, these decisions may result in unnecessary reduced chemotherapy delivery in some older and obese patients [28,29] due to pharmacokinetics with variability in drug metabolism between patients receiving the same chemotherapy regimens [30].

We investigated patient-related characteristics associated with treatment modifications and found that comorbidity increased the odds of both dose reductions, discontinuation and RDI < 85%. This has been reported previously in patients receiving ACT [10,13]. Taking long-term prescription medications at diagnosis was associated with dose reductions and discontinuation with the strongest associations if taking ≥5 different medications. Although this is not surprising since taking medications is expected to be correlated with comorbidity, it is a new important discovery. However, our study was not powered to make sub-analyses of specific chronic diseases or prescription drugs. Although most patients in our study did not have comorbidity and took no or few medications, the significant associations indicate a subgroup with moderate-severe comorbidity possibly being particularly vulnerable to reduced chemotherapy delivery. Furthermore, being overweight (BMI 25–29.9 kg/m^2^) was associated with discontinuation in our study as also reported previously [10,31]. The fact that this may be due to increased toxicity (considering that discontinuation was most frequently related to neuropathy), stands in contrast to other findings of patients with a high BMI not experiencing increased toxicity compared to normal-weight patients [29]. Patients aged ≥ 65 years in our study were three times more likely to receive an RDI < 85% (95% CI 1.1–8.7) compared to younger patients. This confirms age ≥ 65 years as a common variable associated with reduced dose intensity across cancer types [10,31] although others showed that patients with breast cancer aged ≥ 65 years were able to maintain an RDI ≥85% of ACT [13,26]. Positive lymph nodes at diagnosis were statistically significantly associated with dose delay with no obvious explanation and thus it may be a chance finding in our small sample size. One possible cause could be the oncologists preferring to go for full dose chemotherapy for these patients—accepting risk of delay rather than dose reduction. We found no additional statistically significant associations of patient-related characteristics, which may in part be due to low statistical power.

After NACT, our study showed rCR or pCR in around 35% of patients, ranging from 12–83% depending on breast cancer subtype. These response rates correspond to previously shown rates of 24–45% for rCR [32,33] and 8–39% for pCR [16,32,33,34] in patients with breast cancer receiving anthracycline and taxane-based regimens. In agreement with a previous superior response to NACT of HER2+ or triple negative tumours [33,34,35], our findings showed a tendency of both rCR and pCR being most frequent in the patients with subtype ER-HER2+ followed by patients with subtype ER-HER2 normal (triple negative). In contrast, patients with subtype ER+HER2 normal appeared to have the least frequent rates of rCR and pCR (17% and 12%, respectively). These findings are consistent with the results from a retrospective analysis of patients with breast cancer from eight institutions in the United States [33], but they should be interpreted with caution as statistically significant differences were not examined in our study. Of notice, we found no statistically significant differences in rCR or pCR by RDI irrespective of subtype. Although this finding most likely may be attributed to the small sample size, it still indicates that there may be patients for which the RDI < 85% may not be harmful, at least not in terms of tumor response.

This study had important limitations to be mentioned and included in the interpretation of our results. First, it was a retrospective design, which may limit data accuracy. Secondly, patients were included from a single center resulting in a small sample size with small strata of sub-analyses causing wide CIs and perhaps a lack of statistical power to detect differences. Thirdly, this study did not include data on recurrence and survival, which could have further elaborated our findings. Although the findings may not be representative of all patients with breast cancer receiving NACT, we present recent real-world data of NACT delivery and response based on a population-based sample of patients treated in a university hospital covering a Danish region.

Our findings of reduced RDI of NACT in agreement with previous results of ACT confirm a need for further investigations of supportive care initiatives beyond the medical treatment of traditional toxicity symptoms to improve patients’ treatment tolerability. A recent review of strategies to optimize chemotherapy dose intensity recommends increased focus of more consistent G-CSF use according to guidelines [29]. Systematic use of patient reported outcomes (PRO) to assess toxicity in daily clinical practice may also improve symptom monitoring and patient-physician communication related to symptom severity during chemotherapy, which may help prevent unplanned dose reductions and discontinuation [36]. In a randomized controlled trial with 766 patients with metastatic cancer, the use of PROs during chemotherapy showed improved health-related quality of life, fewer hospitalizations and the ability to tolerate chemotherapy longer compared to usual care [37]. Other research suggest that systematic pre-treatment measurements of inflammatory markers may help identify patients at risk for reduced RDI. Although this still needs further investigation, exploratory analysis from a randomized controlled trial showed that having increased inflammatory markers of interleukin (IL)-8 and Tumor Necrosis Factor-alpha (TNF-α) at diagnosis was associated with an RDI < 85% in patients with breast cancer [38]. Physical exercise may be hypothesized to improve chemotherapy delivery in patients with breast cancer, but it would require further investigations due to inconsistent current evidence. According to a systematic review from 2019 [39], two randomized controlled trials found beneficial effects of exercise during ACT on dose intensity compared to usual care [40,41] but as secondary analyses and with methodological issues in outcome definitions. Later, the Opti-Train trial found no beneficial effects of moderate-high intensity exercise on ACT completion rates [42].

Future research using prospectively collected data from larger heterogeneous cohorts should validate our findings and further elaborate on the differences of NACT delivery and treatment response in subgroups by tumour- and patient-related characteristics. Only with detailed data on chemotherapy delivery and tumour response, vulnerable patients can be identified, which is the first step towards developing and testing tailored strategies to improve treatment tolerability and, ultimately, patient outcomes.

## 5. Conclusions

This study showed routine clinical practice patterns of NACT and tumour response in patients with breast cancer. Many patients experienced treatment modifications with dose reductions, dose delays and early discontinuation. While most patients had an RDI ≥85%, still one out of four patients received an RDI < 85%. There is a need for further investigations of possible supportive care initiatives to improve patients’ treatment tolerability, particularly among subgroups of older age or with comorbidity who may be particularly susceptible to receive a suboptimal RDI.

## Figures and Tables

**Table 1:** Patient-related and clinical characteristics at diagnosis of 122 female patients with breast cancer starting neoadjuvant chemotherapy between 2017 and 2019.

	n	%	Median (Range)
**Age (years)**			
All			50 (25–78)
25–49	54	(44)	
50–64	44	(36)	
65–78	24	(20)	
**Cohabitation status**			
Living with partner	85	(70)	
Living alone	37	(30)	
**Work market affiliation**			
Working	71	(58)	
Not working due to illness	19	(15)	
Age retirement	29	(24)	
Missing data	3	(2)	
**Body Mass Index (kg/m^2^)**			
All			26 (17–45)
Under- and normal weight (<25)	53	(44)	
Overweight (25–29.9)	39	(32)	
Obese (≥30)	30	(24)	
**Body surface area for chemotherapy dosing (m^2^)**			
All			1.9 (1.5–2.5)
<2.0	88	(72)	
≥2.0	34	(28)	
Doses capped at 2.0 m^2^ (yes, no)	8, 114	(7, 93)	
**Smoking status**			
Never smoker	56	(46)	
Previous smoker	27	(22)	
Current smoker	31	(25)	
Missing data	8	(6)	
**Alcohol consumption**			
No consumption	52	(43)	
Any consumption	52	(43)	
Missing data	18	(15)	
**WHO Performance status**			
0	114	(93)	
1–2	8	(7)	
**Kidney function (eGFR)**			
<90 mL/min/1.73 m^2^	41	(34)	
≥90 mL/min/1.73 m^2^	81	(66)	
**Comorbidity (chronic diseases)**			
None *	81	(66)	
1	32	(26)	
≥2	9	(7)	
**Long-term medications**			
None *	54	(44)	
1–4 different medications	54	(44)	
≥5 different medications (polypharmacy)	14	(11)	
**Menopausal status**			
Premenopausal	57	(47)	
Postmenopausal	65	(53)	
**Breast cancer subtype**			
ER+HER2 normal	59	(48)	
ER+HER2+	20	(16)	
ER-HER2+	20	(16)	
ER-HER2 normal	23	(19)	
**Positive lymph node involvement**			
None	40	(33)	
Suspected	13	(11)	
Diagnosed by MRI and/or biopsy	69	(56)	
**Planned chemotherapy treatment regimen**			
EC (3 cycles) + PAC (3 cycles)	59	(48)	
EC (4 cycles) + PAC (4 cycles)	61	(50)	
Other/part of a regimen	2	(2)	
**Concomitant HER2 blockade (trastuzumab and pertuzumab)**			
Yes	40	(33)	
No	82	(67)	

Percentages are rounded to whole numbers. * The discrepancy between number of patients with ‘No comorbidity’ and ‘No long-term medications’ is due to hypertension considered as a risk-condition and not a chronic disease, while antihypertensive drugs were counted as long-term drugs. Abbreviations: ER, estrogen receptor; HER2, human epidermal growth factor receptor 2; EC, epirubicin (90 mg/m^2^) and cyclophosphamide (600 mg/m^2^) administered with 3-week intervals; PAC, paclitaxel (80 mg/m^2^) administered weekly; MRI, magnetic resonance imaging; eGFR, estimated Glomerular Filtration Rate.

**Table 2:** Relative dose intensity of neoadjuvant chemotherapy in 122 patients with breast cancer between 2017 and 2019.

	n	%	Median (Range)
**Relative Dose Intensity**			
All	122	(100)	95.23 (8.33–100)
<70%	11	(9)	
70 to 84.99%	19	(16)	
85 to 94.99%	29	(24)	
95 to 99.99%	28	(23)	
100%	35	(28)	

**Table 3:** Uni- and multivariate odds ratios with corresponding 95% confidence intervals (CI) for treatment modifications and suboptimal relative dose intensity of neoadjuvant chemotherapy (RDI < 85%) among 122 female patients with breast cancer between 2017 and 2019.

	Dose Reductions (Yes vs. No)		Dose Delays (Yes vs. No)		Discontinuation (Yes vs. No)		RDI (<85% vs. ≥85%)	
	OR (95% CI)							
Covariate	Crude	Adjusted ^a^	Crude	Adjusted ^a^	Crude	Adjusted ^a^	Crude	Adjusted ^a^
**Age in years**								
25–64	Ref.	Ref.	Ref.	Ref.	Ref.	Ref.	Ref.	Ref.
65–78	3.3 (1.3–8.4) *	2.4 (0.9–6.8)	1.0 (0.4–2.4)	1.1 (0.4–3.1)	1.4 (0.5–3.6)	1.2 (0.4–3.3)	3.5 (1.4–9.0) *	3.0 (1.1–8.7) *
**Cohabitation status**								
Living with partner	Ref.	Ref.	Ref.	Ref.	Ref.	Ref.	Ref.	Ref.
Living alone	1.6 (0.7–3.4)	1.5 (0.6–3.7)	0.6 (0.2–1.3)	0.7 (0.3–1.6)	0.8 (0.3–1.9)	0.7 (0.3–1.7)	1.2 (0.5–2.9)	1.1 (0.4–3.0)
**Work market affiliation**								
Working	Ref.	Ref.	Ref.	Ref.	Ref.	Ref.	Ref.	Ref.
Not working due to illness	2.5 (0.9–6.9)	1.8 (0.6–5.4)	1.4 (0.5–3.8)	1.3 (0.4–3.9)	2.5 (0.8–7.3)	2.3 (0.8–6.9)	1.8 (0.5–6.3)	1.5 (0.4–5.3)
Age retirement	4.9 (1.9–12.6) *	1.1 (0.2–4.8)	1.4 (0.6–3.4)	2.7 (0.6–12.2)	1.8 (0.7–4.7)	1.0 (0.2–4.8)	5.4 (2.0–14.5) *	2.3 (0.4–11.2)
**Body Mass Index ^b^**								
Underweight/Normal	Ref.	Ref.	Ref.	Ref.	Ref.	Ref.	Ref.	Ref.
Overweight	1.2 (0.5–2.8)	1.7 (0.6–4.4)	0.6 (0.2–1.4)	0.8 (0.3–1.9)	2.6 (1.0–6.7) *	2.8 (1.1–7.4) *	1.2 (0.5–3.0)	1.6 (0.6–4.4)
Obese	0.7 (0.3–1.9)	1.5 (0.5–4.4)	0.4 (0.1–1.0)	0.7 (0.2–2.1)	1.2 (0.4–3.4)	1.4 (0.4–4.4)	0.4 (0.1–1.4)	0.8 (0.2–2.9)
**Body surface area**								
<2 m^2^	Ref.	Ref.	Ref.	Ref.	Ref.	Ref.	Ref.	Ref.
≥2 m^2^	1.2 (0.5–2.7)	2.0 (0.8–5.0)	1.0 (0.4–2.1)	1.5 (0.6–3.6)	2.0 (0.8–4.6)	2.2 (0.9–1.1)	1.1 (0.5–2.8)	1.7 (0.6–4.9)
**Comorbidity**								
None	Ref.	Ref.	Ref.	Ref.	Ref.	Ref.	Ref.	Ref.
1	3.3 (1.4–7.8) *	3.4 (1.3–8.9) *	1.3 (0.6–3.0)	1.7 (0.7–4.2)	0.8 (0.3–2.1)	0.7 (0.3–2.0)	1.3 (0.5–3.6)	1.1 (0.4–3.2)
≥2	4.0 (0.9–17.2)	2.1 (0.4–11.0)	1.2 (0.3–4.9)	1.4 (0.3–6.8)	5.7 (1.3–24.9) *	4.8 (1.0–22.8) *	8.1 (1.8–36.0) *	5.1 (1.0–26.4) *
**Long term medications**								
None	Ref.	Ref.	Ref.	Ref.	Ref.	Ref.	Ref.	Ref.
1–4 different medications	4.4 (1.9–10.0) *	4.1 (1.6–10.8) *	0.6 (0.3–1.3)	0.5 (0.2–1.3)	1.7 (0.7–4.2)	1.8 (0.7–4.6)	1.2 (0.5–3.2)	0.8 (0.3–2.4)
≥5 different medications	12.8 (3.0–53.5) *	8.4 (1.8–39.9) *	1.5 (0.5–5.0)	1.6 (0.4–6.4)	7.9 (2.2–28.8) *	8.2 (1.9–34.3) *	5.9 (1.7–20.7) *	3.0 (0.7–12.5)
**Breast cancer subtype**								
ER+HER2 normal	Ref.	Ref.	Ref.	Ref.	Ref.	Ref.	Ref.	Ref.
ER+HER2+	1.0 (0.4–2.9)	0.6 (0.2–2.0)	1.9 (0.7–5.4)	1.4 (0.4–4.3)	2.4 (0.8–6.9)	2.3 (0.8–6.8)	3.3 (1.1–10.0) *	2.4 (0.7–8.2)
ER-HER2+	1.0 (0.4–2.9)	1.5 (0.4–4.8)	1.9 (0.7–5.4)	1.3 (0.4–4.3)	0.7 (0.2–2.5)	0.8 (0.2–3.1)	1.2 (0.3–4.4)	1.6 (0.4–6.8)
ER-HER2 normal	0.8 (0.3–2.2)	0.9 (0.3–2.7)	1.8 (0.7–4.8)	1.5 (0.5–4.2)	1.0 (0.3–3.1)	1.1 (0.4–3.4)	2.6 (0.9–7.8)	3.4 (0.9–11.1)
**Tumour size**								
<50 mm	Ref.	Ref.	Ref.	Ref.	Ref.	Ref.	Ref.	Ref.
≥50 mm	2.2 (0.7–6.2)	1.5 (0.5–4.9)	1.9 (0.6–5.3)	1.7 (0.5–5.2)	1.2 (0.4–3.7)	1.0 (0.3–3.4)	3.1 (1.0–9.5) *	2.3 (0.7–7.6)
**Positive lymph node involvement ^c^**								
No	Ref.	Ref.	Ref.	Ref.	Ref.	Ref.	Ref.	Ref.
Yes	1.4 (0.7–3.1)	1.3 (0.5–3.0)	2.1 (0.9–4.7)	2.3 (1.0–5.6) *	1.5 (0.6–3.6)	1.4 (0.6–3.5)	1.5 (0.6–3.7)	1.3 (0.5–3.5)

* *p*-value < 0.05. ^a^ Adjusted for age (continuous), estimated Glomerular Filtration Rate (eGFR) (<90, ≥90 mL/min/1.73 m^2^) and granulocyte-colony stimulating factor (G-CSF) administered during neoadjuvant chemotherapy (yes, no). ^b^ Body Mass Index: Underweight (<18.5 kg/m^2^)/Normal (18.5–24.9 kg/m^2^), Overweight (25–29.9 kg/m^2^), Obese (≥30 kg/m^2^). ^c^ Positive lymph node involvement: None, Suspicion/Diagnosed by magnetic resonance imaging and/or biopsy. Abbreviations: OR, odds ratio; CI, confidence interval; RDI, relative dose intensity; ER; estrogen receptor, HER2; human epidermal growth factor receptor 2.

**Table 4:** Tumour response by magnetic resonance imaging and surgically excised breast tissue after neoadjuvant chemotherapy in 118 patients with breast cancer between 2017 and 2019, by subtype and relative dose intensity.

	rCR		Non-rCR			pCR (Grade I)		Non-pCR (Grade II–IV)		
	n	(%)	n	(%)	*p*-Value *	n	(%)	n	(%)	*p*-Value *
**All (n = 118)**	43	(36)	75	(64)	0.35	41	(35)	75	(65)	>0.99
RDI ≥ 85% (n = 92)	36	(39)	56	(61)		32	(35)	58	(65)	
RDI < 85% (n = 26)	7	(27)	19	(73)		9	(35)	17	(65)	
ER+HER2 normal (n = 59) **	10	(17)	49	(83)	>0.99	7	(12)	50	(88)	>0.99
RDI ≥ 85% (n = 49)	9	(18)	40	(82)		6	(13)	41	(87)	
RDI < 85% (n = 10)	1	(10)	9	(90)		1	(10)	9	(90)	
**ER+HER2+ (n = 20)**	10	(50)	10	(50)	0.65	9	(45)	11	(55)	>0.99
RDI ≥ 85% (n = 12)	7	(58)	5	(42)		5	(42)	7	(58)	
RDI < 85% (n = 8)	3	(38)	5	(62)		4	(50)	4	(50)	
**ER-HER2 normal (n = 21)**	9	(43)	12	(57)	0.65	10	(48)	11	(52)	0.63
RDI ≥ 85% (n = 15)	7	(47)	8	(53)		8	(53)	7	(47)	
RDI < 85% (n = 6)	2	(33)	4	(67)		2	(33)	4	(67)	
**ER-HER2+ (n = 18)**	14	(78)	4	(22)	0.40	15	(83)	3	(17)	>0.99
RDI ≥ 85% (n = 16)	13	(81)	3	(19)		13	(81)	3	(19)	
RDI < 85% (n = 2)	1	(50)	1	(50)		2	(100)	0	(0)	

* *p*-value from Fisher’s exact test. ** Two patients with subtype ER+HER2 normal had missing data on pathologic response grades giving a total of 116 patients. Abbreviations: rCR, radiologic complete response: tumour size = 0 mm/disappearance of all lesions by magnetic resonance imaging; non-rCR, radiologic non-complete response: tumour size > 0 mm/residual tumour by magnetic resonance imaging; pCR, pathological complete response with no remaining malignant cells in surgically excised breast tissue; non-pCR, remaining malignant cells in surgically excised breast tissue; RDI, relative dose intensity.

## Data Availability

The data are not publicly available due to research participant privacy restrictions.

## Supplementary Materials

The following supporting information can be downloaded at: https://www.mdpi.com/article/10.3390/cancers15092483/s1, Table S1: Comorbidity and long-term medications at diagnosis among 122 female patients with breast cancer starting neoadjuvant chemotherapy between 2017 and 2019; Figure S1: Planned and received neoadjuvant chemotherapy among 30 female patients with breast cancer with a relative dose intensity (RDI) below 85% between 2017 and 2019.
