# Supplementary material for: Patient-Related Characteristics Associated with Treatment Modifications and Suboptimal Relative Dose Intensity of Neoadjuvant Chemotherapy in Patients with Breast Cancer—A Retrospective Study
Source: Cancers (Basel). 2023 Apr 26;15(9):2483. doi: 10.3390/cancers15092483 (PMC10177586; doi:10.3390/cancers15092483)
Supplement: Supplementary file 1 [file cancers-15-02483-s001.zip › cancers-2243982-supplementary.pdf]

Table S1. Comorbidity and long-term medications at diagnosis among 122 female patients with breast cancer starting neoadjuvant chemotherapy between 2017 and 2019.

	N	%
**Comorbidity (chronic diseases)**		
No comorbidity	81	(66)
1	32	(26)
≥ 2	9	(7)
Coronary heart disease	1	(1)
Peripheral vascular disease	2	(2)
Chronic obstructive pulmonary disorder	4	(3)
Diabetes	6	(5)
Thyroid disorders	11	(9)
Kidney disease	2	(2)
Inflammatory bowel disease	2	(2)
Ulcer	2	(2)
Hemiplegia/stroke	5	(4)
Multiple sclerosis	1	(1)
Other previous cancer	4	(3)
Dementia	1	(1)
Rheumatoid and connective tissue	2	(2)
Depression/anxiety	10	(8)
Psychotic diseases	1	(1)
**Long-term medications**		
None	54	(44)
1-4 different medications	54	(44)
≥ 5 different medications	14	(11)
Antihypertensive drugs	32	(26)
Antithrombotic drugs	10	(8)
Antihyperlipidemic drugs	14	(11)
Diuretics	3	(2)
Bronchodilators	14	(11)
Analgesics	20	(16)
Proton-pump inhibitors	13	(11)
Antidiabetics	6	(5)
Drugs for thyroid disorders	11	(9)
Drugs for osteoporosis	1	(1)
Drugs for constipation/incontinence	3	(2)
Hypnotics	5	(4)
Antidepressants/anxiolytics/antipsychotics	13	(11)
Drugs for dementia	1	(1)
Other drugs	4	(3)

Percentages are rounded to whole numbes

Patient	Cycle 1	Cycle 2	Cycle 3	Cycle 4	Cycle 5	Cycle 6	Cycle 7	Cycle 8	Delay (days)	RDI (%)
1	100								0	8.3
2	100								0	16.7
3	100								0	22.2
4	100	100							0	33.3
5	100	100	75						0	46.1
6	50	75	75	75	75 75 75	75 75 75	75 75 75	75 75 75	13	63.8
7	75	75	75	80 100 100	100 100				7	66.0
8	75	75	75	75	75 75 75	75 75 75	60 60 60	60 60	15	67.3
9	75	75	75 75 75	75 75 75	75 67 67	67 67 67	67 67 67	67 67	17	68.5
10	75	75	75	75 75 75	75 75 75	75 75 75	75 75 75	75 75 75	13	68.8
11	100	100	50	100	100 100 100	100 100 100	100 100		7	70.3
12	100	100	0,5	100 100 100	100				0	70.3
13	75	75	75	100 100 100	100 100				7	72.6
14	100	100	100 100 100	100					0	74.1
15	100	100	75	100 100 100	75 75 75				30	74.7
16	100	100	100	100	100 100 100				0	75.0
17	75	75	75	75	75 100 100	100 100 100	100 100 100	100 100 100	13	75.9
18	100	100	100	100	100 100 80	80			11	76.0
19	75	75 75 75	75 75 75	75 75 75	75 75 75				0	77.1
20	100	100	100	100 100 100	75 75 75	75			19	77.5
21	100	100	75	75	100 100 100	100 100 100	100 100 100	100 100 100	32	77.8
22	100	100	90	90	100 100 100	90 90 90			4	78.5
23	100	100	100	100 100 100	100 100 100	100 100 75			8	80.3
24	100	100	100	100	100 100 100	100 100 100	100 100 100		21	81.2
25	100	100	100	75	100 100 100	100 100 100	100 100 100	100 100 100	14	82.9
26	100	100	100 100 100	100 100 100					0	83.3
27	100	100	100	100	100 100 100	100 100 100			0	83.3
28	75	75	75	75	100 100 100	100 100 100	100 100 100	100 100 100	0	83.3
29	75	75	75	100 100 100	100 100 100	100 100 100			0	83.3
30	100	100	100	100 100 100	100 100 100	100			10	83.4

Received % of standard doses epirubicin (90 mg/m^2) and cyclophosphamide (600 mg/m^2)

Received % of standard dose paclitaxel (80 mg/m^2)

Planned doses not received due to discontinuation

Note: If there was change in regimen (patient 9, 10, 13, 14, 15, 19, 23, 26), the second planned treatment based on the Danish Breast Cancer Cooperative Group guidelines was considered the standard total dose.

Figure S1. Planned and received neoadjuvant chemotherapy among 30 female patients with breast cancer with a relative dose intensity (RDI) below 85% between 2017 and 2019.
